# Recombinant linear multiple epitopes of σB protein protect Muscovy ducks against novel duck reovirus infection

**DOI:** 10.3389/fvets.2024.1360246

**Published:** 2024-05-13

**Authors:** Yiquan Chen, Zhuanqiang Yan, Changtao Liao, Yiwei Song, Qi Zhou, Hanqin Shen, Feng Chen

**Affiliations:** ^1^College of Animal Science, South China Agricultural University, Guangzhou, China; ^2^Guangdong Enterprise Key Laboratory for Animal Health and Environmental Control, Wen's Foodstuff Group Co. Ltd., Yunfu, China

**Keywords:** novel duck reovirus (NDRV), σB protein, linear B cell epitopes, vaccine, immunization

## Abstract

Infection by the novel duck reovirus (NDRV) in ducklings causes high mortality, which leads to substantial economic losses in the duck industry in China. To date, no commercial vaccine is available for this disease. In this study, linear B cell epitopes of the σB protein of the NDRV were predicted and recombinant multiple linear B cell epitopes (MLBEs) were constructed through linkers. The recombinant MLBEs were then expressed and purified. One-day-old Muscovy ducklings were immunized with different doses of MLBEs and challenged with 5 × 10^4^ ELD_50_ of the virulent CHY strain of NDRV 14 days after immunization. The ducklings vaccinated with 20 and 40 μg of MLBE performed no clinical signs or gross or histopathological lesions, indicating 100% protection against infection. The viral load in the liver and spleens of these birds was significantly lower than that in the control group. Additionally, these ducklings exhibited positive seroconversion at 7 days after vaccination on enzyme-linked immunosorbent assay. These results indicate that MLBE of σB could be used as a candidate for developing vaccines against NDRV infection.

## 1 Introduction

Avian orthoreoviruses (ARVs), belonging to the genus *Orthoreovirus* of the family *Spinareoviridae*, are critical etiological agents that can cause devastating diseases in various domestic and wild birds (1). ARVs can cause viral arthritis/tenosynovitis and are associated with several diseases in chickens, such as respiratory disease, enteric disease, and inclusion body hepatitis (2, 3). Muscovy duck reovirus (MDRV) infection mainly causes liver foci necrosis, resulting in high mortality in 1-day-old Muscovy ducklings (4, 5). Similar symptoms and 10–20% mortality have been recently reported in geese after ARV infection (6). Since 2011, a novel duck reovirus (NDRV) infection among different duck species, such as Peking, mallard, and Muscovy ducks, has caused severe hemorrhagic–necrotic lesions in the liver and spleen, resulting in 5–50% mortality in ducklings, which is different from MDRV infection (7–11). ARV infection has caused significant economic losses for the poultry industry in China.

The ARV genome consists of 10 double-stranded RNA segments, divided into three size classes: large (L1–L3), medium (M1–M3), and small (S1–S4) (1, 12), which encode at least 12 proteins. Sigma C and σB are the major surface proteins encoded by the S1 and S3 gene segments, respectively (13). Both sigma C and σB can induce specific neutralizing antibodies; hence, they are targets for vaccine development or disease diagnosis (13–16).

Vaccination is a major strategy for the prevention and control of infectious diseases. One commercial MDRV-CA strain live attenuated vaccine, is available to prevent the disease in China. However, it does not provide cross-protection against NDRV infection (17, 18). Therefore, developing an efficient vaccine to control NDRV infection is urgently needed. A naturally attenuated NDRV N20 strain provided sufficient protection (19), while a subunit vaccine consisting of sigma C protein produced in Sf9 cells induced 100% protection against the challenge with lethal NDRV (20).

In this study, the predicted linear B cell epitopes fused with sumo-tags were expressed in *Escherichia coli*. The humoral immune responses and protection provided by recombinant multiple linear B cell epitopes (rMLBEs) against NDRV challenge were evaluated in Muscovy ducklings, which can be used a reference for NDRV vaccine development.

## 2 Materials and methods

### 2.1 Ethics statement

The protocol was approved by the Committee of Animal Experiments of South China Agricultural University, Guangzhou, China (Approval ID: SYXK-2019-0136). All biological safety and sanitation measures were observed.

### 2.2 B cell epitope prediction and protein expression

The linear B cell epitopes of σB protein (TH11 strain, NCIB accession No. AFX68863.1) were predicted using Bepipred Linear Epitope Prediction 2.0 (http://tools.iedb.org/bcell/) (21). The multiple-epitope subunit vaccine was constructed using the B-cell epitopes through a GGGGG linker, and codon optimization was achieved using the Java Codon adaptation tool (http://www.jcat.de/) to improve its expression level in the BL21 (DE3) strain of *E. coli* (21). The optimized sequence was synthesized by Sangon Biotech (Shanghai, China) and cloned into the pSmart-I vector (Convenience Biology, Changzhou, China) downstream of the *Sumo* gene using the *BamH* I and *Xho* I restriction sites. The recombinant plasmid was used to transform the *E. coli* BL21 (DE3) cells; single colonies were selected and grown in LB medium with 50 μg/mL kanamycin at 37°C until an OD_600_ of 0.5–0.6 was reached. Protein expression was induced by adding isopropyl β-D-thiogalactopyranoside (IPTG) to a final concentration of 0.1 mM and incubated at 16°C overnight. Cells were collected, resuspended in buffer A [20 mM Tris, 250 mM NaCl, 5% (V/V) glycerine; pH ± 8.0], and ultrasonicated. The cell lysate was centrifuged at 13,000 *g* and 4°C for 30 min. The recombinant proteins in the supernatant were purified by nickel-nitrilotriacetate (Ni-NTA) affinity chromatography, as previously described (22). Briefly, the supernatants were loaded on the Ni-NTA agarose by gravity flow and washed with buffer B (20 mM Tris, 250 mM NaCl, 50 mM imidazole; pH 8.0). The protein of interest was eluted with buffer C (20 mM Tris, 250 mM NaCl, 250 mM imidazole; pH ± 8.0). The eluate was dialyzed in PBS buffer (pH 7.4) and analyzed via sodium dodecyl sulfate–polyacrylamide gel electrophoresis (SDS–PAGE) and western blotting.

### 2.3 Animal immunization and challenge

The purified rMLBE protein or PBS was mixed with oil adjuvant to obtain the rMLBE or control vaccines, respectively. For the animal studies, 60 one-day-old NDRV-free Muscovy ducks were supplied by the Wens Food Group Co. Ltd. and randomly divided into four groups of 15 each. Of them, three groups were immunized with 10, 20, and 40 μg of rMLBE vaccine on day 0; while one group was immunized with PBS. Sera from the immunized groups were collected at 0, 7, and 14 days post-vaccination (DPV) for antibody detection. The birds were challenged with 5 × 10^4^ ELD_50_ of the NDRV CHY strain at 14-DPV. Three ducks from each group were sacrificed at 3, 5, and 7 days post-challenge (DPC), and their spleens and livers were collected for viral load determination.

### 2.4 Indirect enzyme-linked immunosorbent assay

The specific IgY antibody titer was determined by indirect enzyme-linked immunosorbent assay (ELISA) using sample sera diluted 100-fold in PBST with 2 mg of *E. coli* lysates and 10 μg of sumo-tag protein. Briefly, 96-well microtiter plates (Costar, USA) were coated with 1 μg/well of purified ΣB protein in 0.1 M carbonate/bicarbonate buffer (pH ± 9.6) and incubated at 4°C overnight. After washing thrice in PBST, the plates were blocked with 5% BSA in PBST for 1 h at 37°C. After washing thrice in PBST, 100 μL of the diluted serum was added to the microtiter wells and incubated for 1 h at 37°C. After washing thrice in PBST, 100 μL of horseradish (HRP)-conjugated anti-duck IgY antibody diluted to 1:3000 in PBST (KPL, USA) was added and incubated for 1 h at 37°C. After washing thrice in PBST, 100 μL of 3,3′,5,5′-Tetramethylbenzidine (TMB) was added to each well and incubated for 10 min at room temperature. The reaction was stopped by adding 50 μL of 2 M H_2_SO_4_, and the OD_450_ was measured. The S/P was calculated using the formula: S/P = (OD test serum-OD negative control serum)/(OD positive control serum-OD negative control serum). The test serum was considered positive when the S/P was > 0.2.

### 2.5 Fluorescent quantitative RT-PCR

Fluorescent quantitative (qRT-PCR) was used to determine the viral load of NDRV in the tissues as described previously (23). The tissues were homogenized, and viral RNA was extracted using MagPure Viral Nucleic Acid Micro LQ Kit (Magen, Guangzhou, China). qRT-PCR was performed as described previously (24) using a One Step RT-qPCR Probe Kit (Yeasen, Shanghai, China) with the forward primer: CCCGGATTCTCGATGAATGGT, reverse primer: CGACCCACTGCTGGATACAAG, and the probe: FAM-AACGCCTGTGCACGAGCTGAAC-3′-TAMRA under the following conditions: 50°C for 15 min, 95°C for 2 min, 40 cycles of 94°C for 15 s, and 60°C for 30 s. The viral load was determined using a standard curve. Each sample was run in triplicates.

### 2.6 Histopathology

Livers and kidneys of the ducks of different groups were collected 3, 5, and 7 DPC and fixed in 10% formalin for 48 h at 25°C. These tissues were embedded in paraffin wax, sliced into 4-μm-thick sections, stained with hematoxylin and eosin (HE), and then examined using light microscopy.

### 2.7 Statistical analysis

Statistical analyses of IgY antibodies were conducted using two-way ANOVA by GraphPad Prism 6 (www.graphpad.com). Statistical analyses of the results of other experiments were performed using an unpaired *t*-test. Differences were considered significant at ^*^p < 0.05, ^**^p < 0.01, or ^***^p < 0.001.

## 3 Results

### 3.1 Expression and purification of the recombinant σB protein

**Figure 1:**
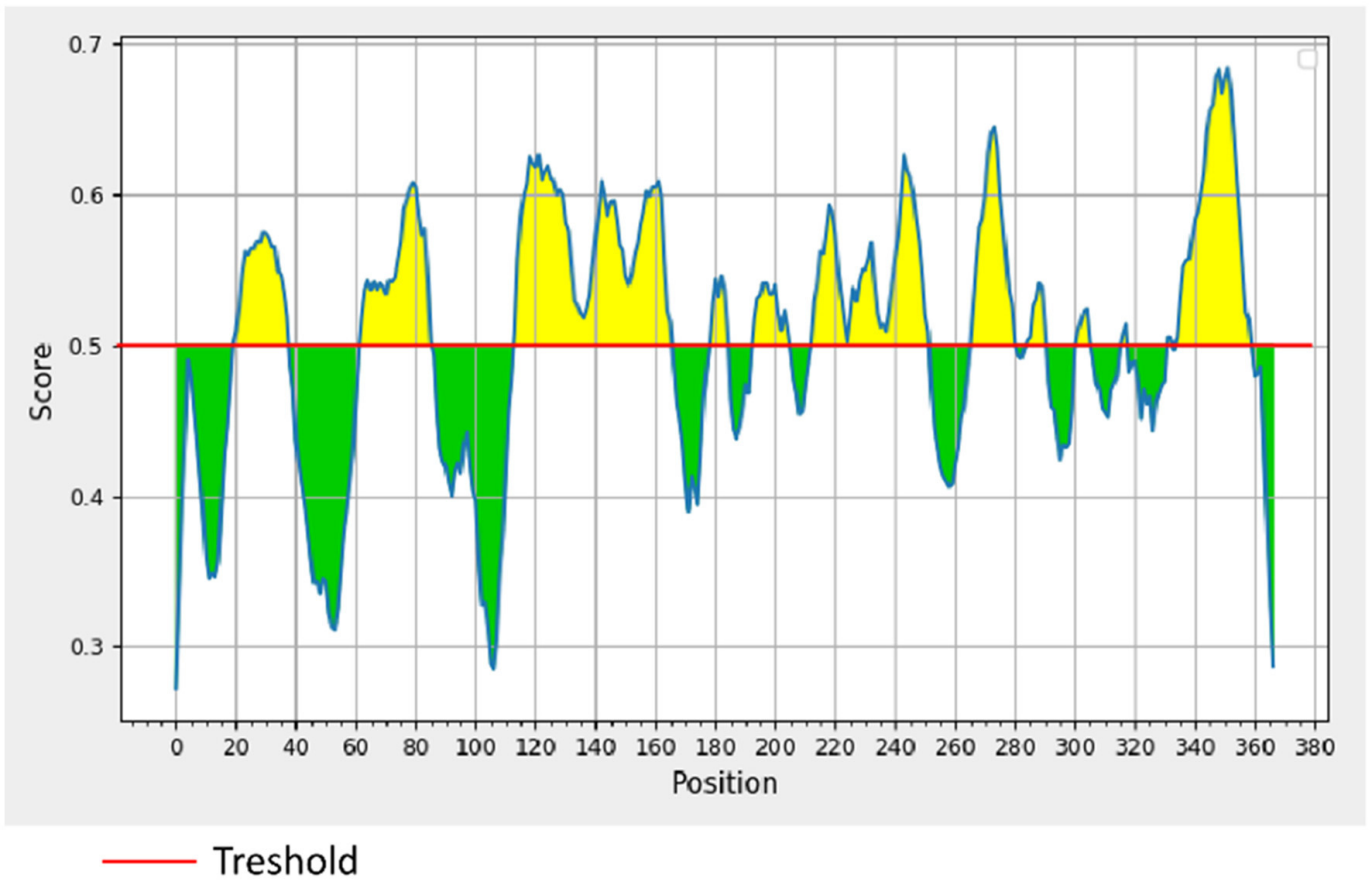
BepiPred linear prediction. Areas above the red line (threshold) are epitopes suggested to be binding to the B cells while the green areas are not.

**Figure 2:**
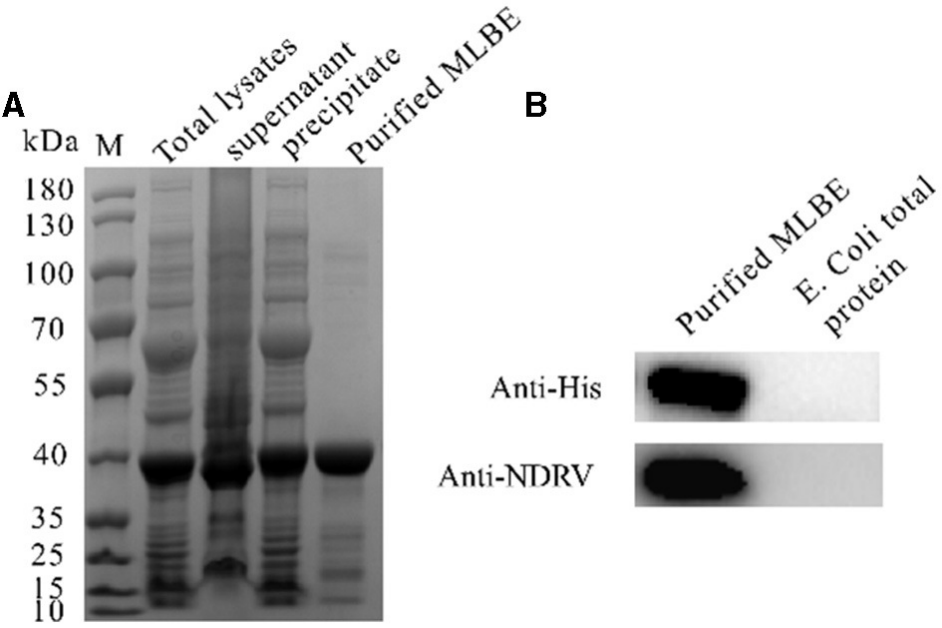
Expression and purification of MLEB of NDRV σB protein **(A)** and western-blotting analysis of the purified MLEB protein **(B)**.

σB is an outer layer protein of NDRV. Therefore, it was proposed that sigma could induce immunological protection. The linear B cell epitopes of σB protein were predicted using Bepipred Linear Epitope Prediction 2.0. When the value was >0.5, the peptides with more than 15 residues were chosen for constructing the multiple linear B cell epitopes. As shown in Figure 1 and Table 1, five epitopes were selected and linked with the flexible peptide “GGGGG.” The expression and purification of MLBE were ascertained by SDS–PAGE and western blotting. As shown in Figures 2A, B, MLBE was successfully expressed and purified.

**Table 1:** Prediction of linear B cell epitopes of σB protein.

**No**.	**Star**	**End**	**Peptide**	**Length**
1	20	38	LCSPACWNSKTLWDIEEFH	19
2	63	86	PPSDGNCFPHHKCHQQQYRTETPL	24
3	114	166	YDEASKQPHDIAETESIAPFDIVTRTESIRSDRAVDPEFWTYPLERRGYDARH	53
4	229	252	PTRGDGAVALSRGNLDHDVEDCWM	24
5	267	281	TGQFERGSCHNFGHP	15
6	335	359	LPDICDFEETTHVGQSSAPLKKATK	25

### 3.2 Humoral immune responses in ducklings immunized with MLBE

**Figure 3:**
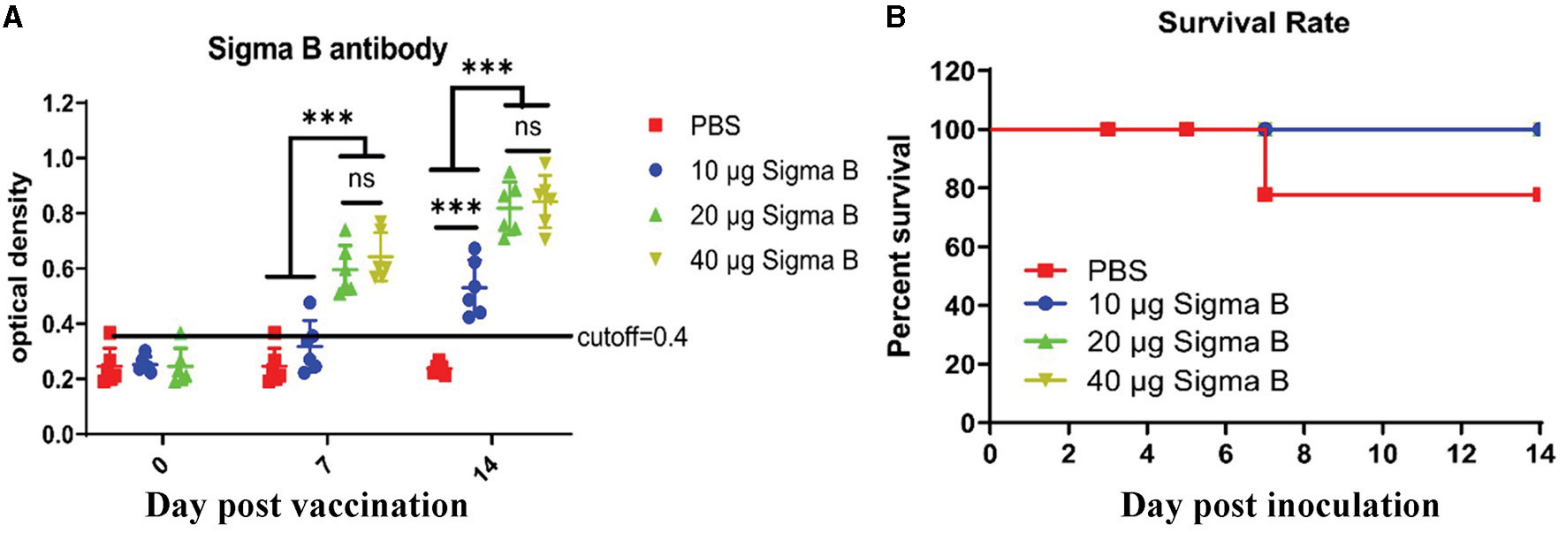
Antibodies analysis **(A)** and survival rate **(B)**. IgY antibodies post MLEB of σB protein were detected by indirect ELISA **(A)**. ns means no significant, *** means *p* < 0.001.

The birds were immunized with different dosages of MLBE, and sera were collected on the indicated dates. NDRV-specific antibodies produced in the birds were detected by indirect ELISA. As shown in Figure 3A, the serum conversion rate of ELISA antibodies was 100% in the 20 and 40 μg immunized birds on 7 DPV and 14 DPV, whereas only one bird was positive among those immunized with 10 μg on 7 DPV. The serum conversion rate was 100% in these birds on 14 DPV. Moreover, the OD_450_ values of 20- and 40-μg immunized birds were significantly higher than the 10 μg-immunized birds on 7 and 14 DPV. However, there was no statistical difference between the 20- and 40 μg-immunized birds. These data indicate that σB MLBE can induce positive immune responses, significantly increasing antibody levels.

### 3.3 Protection of ducklings from the NDRV challenge

**Figure 4:**
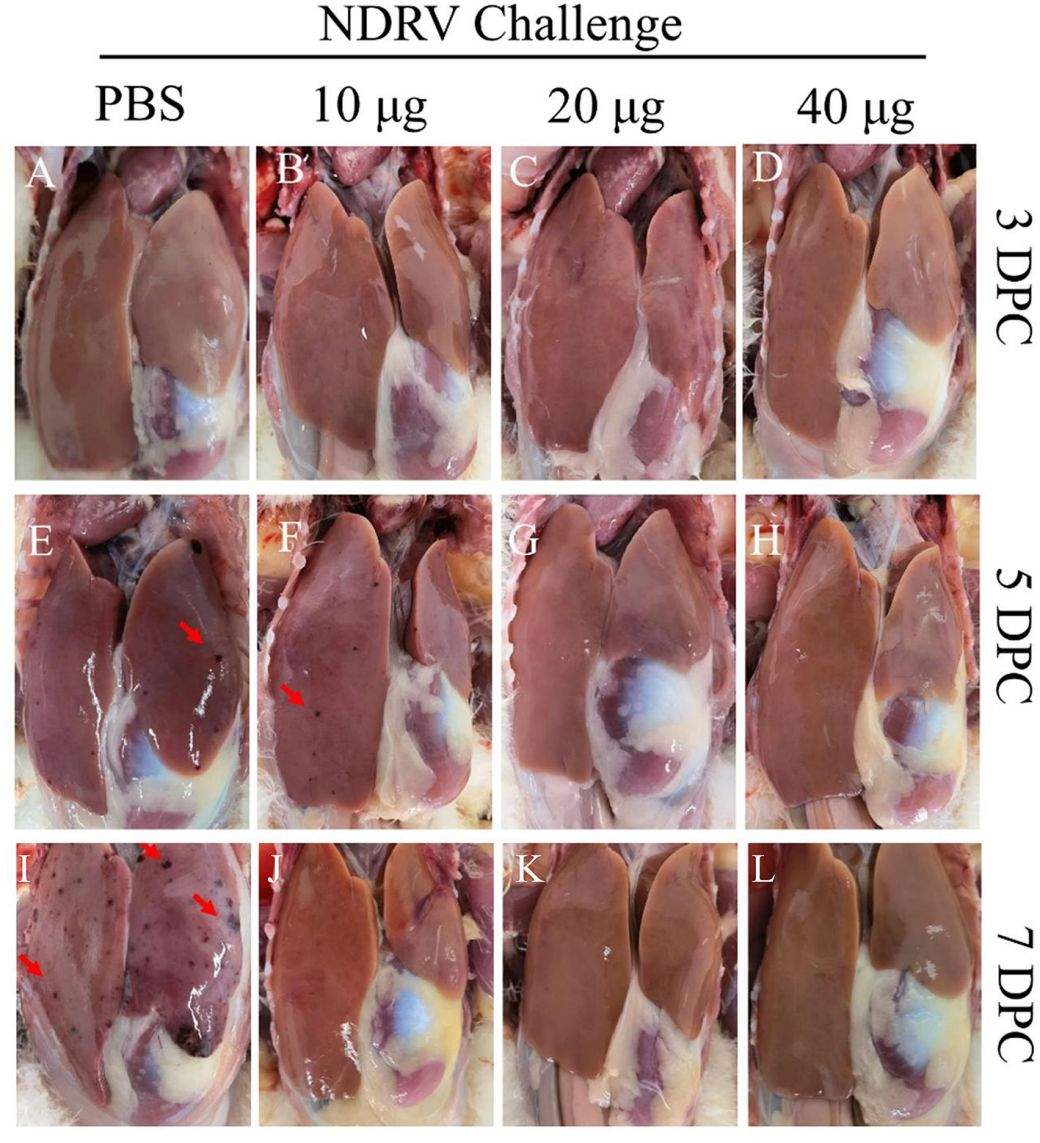
**(A–D)** Clinical changes of the livers on 3 DPC. **(E–H)** Clinical changes of the livers on 5 DPC. **(I–L)** Clinical changes of the livers on 7 DPC. Red arrowheads means Lesion spots.

**Figure 5:**
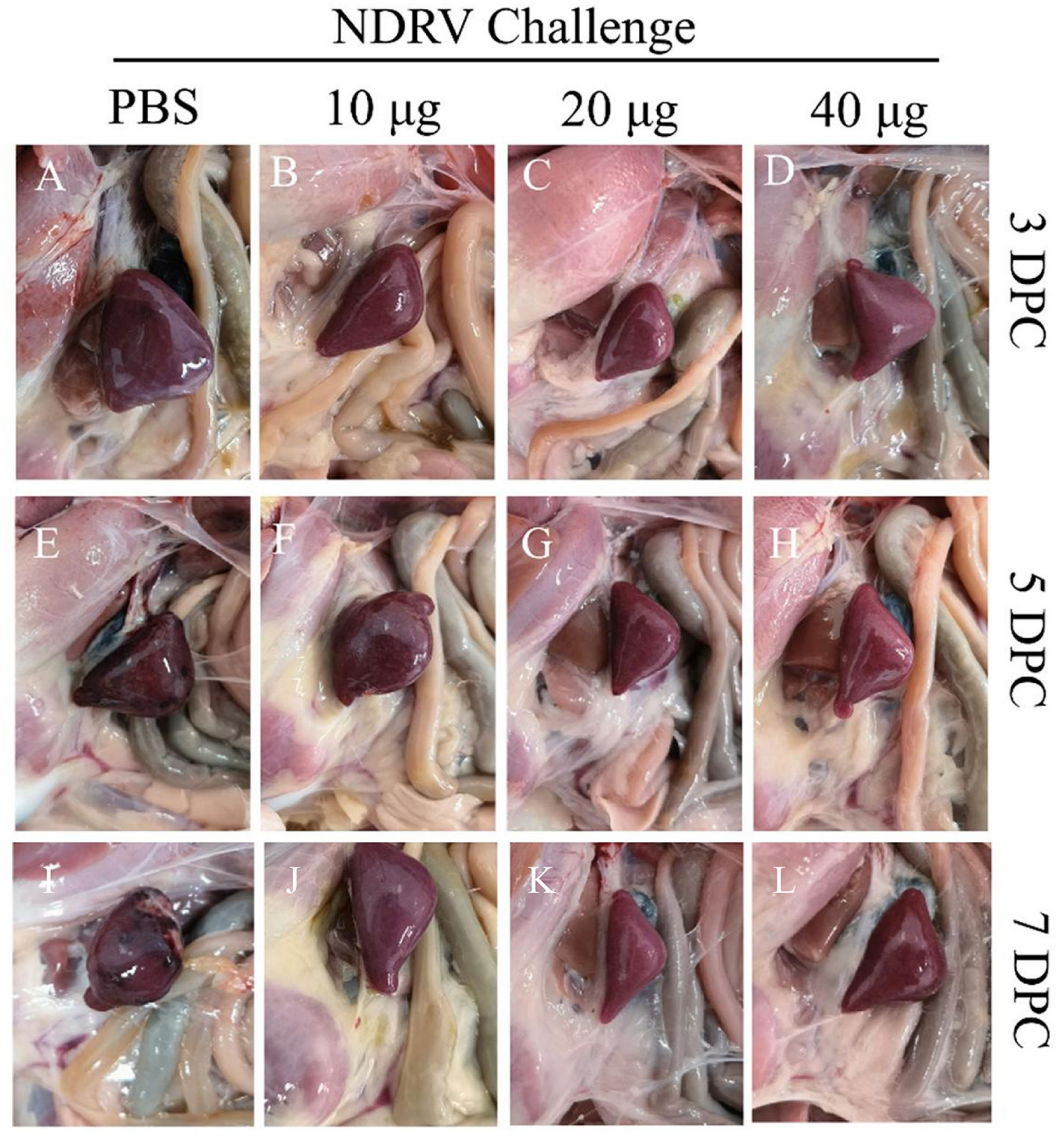
**(A–D)** Clinical changes of the spleens on 3 DPC. **(E–H)** Clinical changes of the spleens on 5 DPC. **(I–L)** Clinical changes of the spleens on 7 DPC.

To assess the protective ability of MLBE, the birds were challenged at 14 DPV, and the clinical symptoms, gross lesions, and histologic microlesions were monitored. At 7 DPC, two birds died in the PBS group (Figure 3B), whereas no birds died during the challenge period in the immunized groups. Three birds were sacrificed for the examination of lesions. The liver and spleen are the primary target organs of NDRV. As shown in Figure 3, slight lesion spots in the liver were observed in the PBS group at 5 DPC (Figure 4E), whereas massive lesion spots were observed at 7 DPC (Figure 4J). Only slight blood spots were observed in the livers of the 10-μg MLBE group at 5 DPC (Figure 4F). No apparent lesions were observed in the livers of the 20- and 40-μg MLBE groups from 3 to 7 DPC (Figure 4). Lesion spots were also observed at 5 and 7 DPC in the PBS group (Figures 5E, J). Otherwise, the spleen was swollen in the 10-μg MLBE group at 5 and 7 DPC (Figures 5F, K). As expected, no noticeable lesions were observed in the spleen of 20- and 40-μg MLBE groups from 3 to 7 DPC (Figure 5).

**Figure 6:**
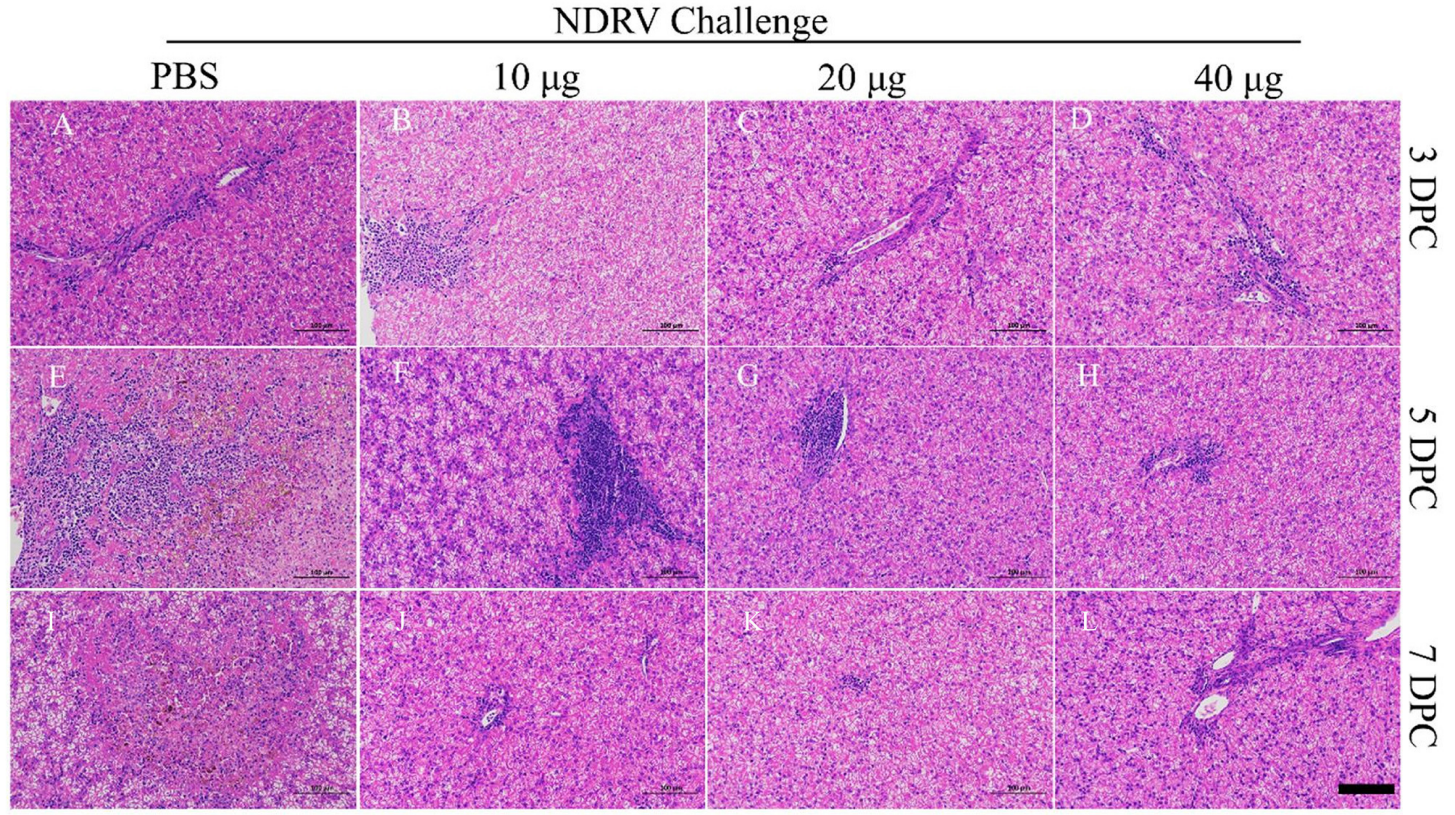
**(A–D)** Histological changes of the livers on 3 DPC. **(E–H)** Histological changes of the livers on 5 DPC. **(I–L)** Histological changes of the livers on 7 DPC.

**Figure 7:**
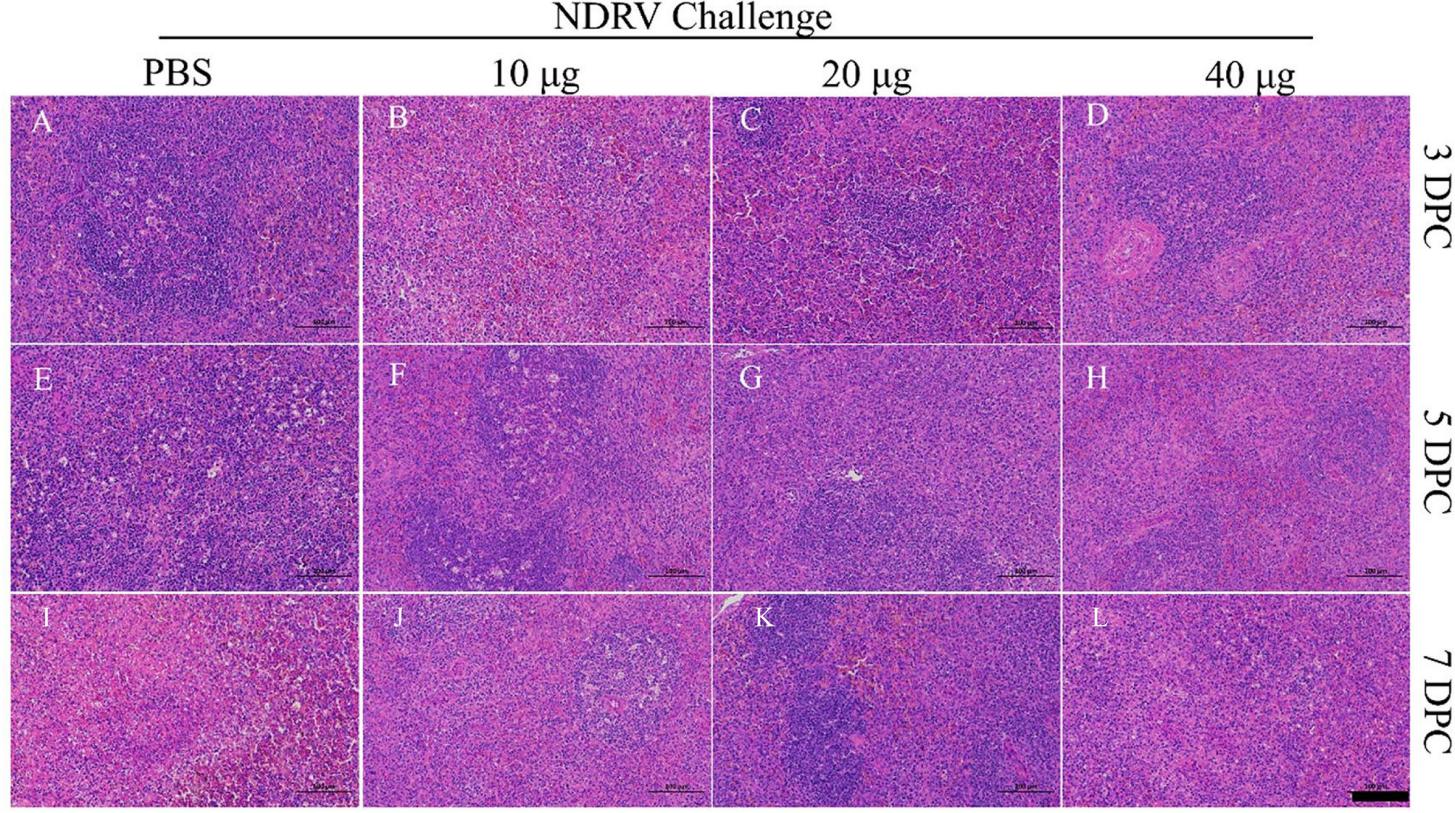
**(A–D)** Histological changes of the spleens on 3 DPC. **(E–H)** Histological changes of the spleens on 5 DPC. **(I–L)** Histological changes of the spleens on 7 DPC.

The corresponding tissues were fixed, sectioned, and stained with HE (Figures 6, 7). Massive pathological damage was observed in the livers of the birds in the PBS group (Figure 6). Necrosis of local hepatocytes and cytolysis were observed at 5 DPC, around which massive inflammatory cell infiltration and hyperpigmentation were evident (Figure 6E). Focal necrosis of hepatocytes and nucleus fragmentation and nucleolysis were also observed at 7 DPC (Figure 6J). No noticeable pathological change was observed in the immunized groups (Figure 6). Massive pathological damage was also observed in the spleens of the PBS group ducks (Figure 7). An enlarged spleen and an unclear boundary were observed at 3 and 5 DPC (Figures 7A, E), whereas massive necrosis with hemorrhage was observed at 7 DPC (Figure 7J). Spleen and lymphatic nodule enlargement were also observed at 5 and 7 DPC in the 10-μg MLBE group (Figures 7F, K), but no evident pathological change was observed in the 20 and 40-μg MLBE groups. These results demonstrated that the MLBE of σB protein protected birds against virulent NDRV-induced pathological changes.

### 3.4 Detection of viral shedding and load in the spleens and livers

From each group, six cloacal swabs were collected at 3, 5, and 7 DPCs for detecting viral shedding using qRT-PCR. As shown in Table 2, viral shedding in a high proportion of birds in the PBS and 10-μg MLBE groups was observed at 5 and 7 DPC. A low proportion of birds in the 20- and 40-μg MLBE groups demonstrated viral shedding at 5 DPC, while only 3/6 birds in the 20 μg and 2/6 birds in the 40-μg MLBE group shed virus particles (Table 2). These results indicated that MLBE can impede viral shedding.

**Table 2:** Detection of virus in the cloacal swabs by qRT-PCR.

**Groups**	**Days post-challenge**		
	**3**	**5**	**7**
PBS	1/6	4/6	6/6
10 μg MLBE	0/6	4/6	5/6
20 μg MLBE	0/6	0/6	3/6
40 μg MLBE	0/6	1/6	2/6

**Figure 8:**
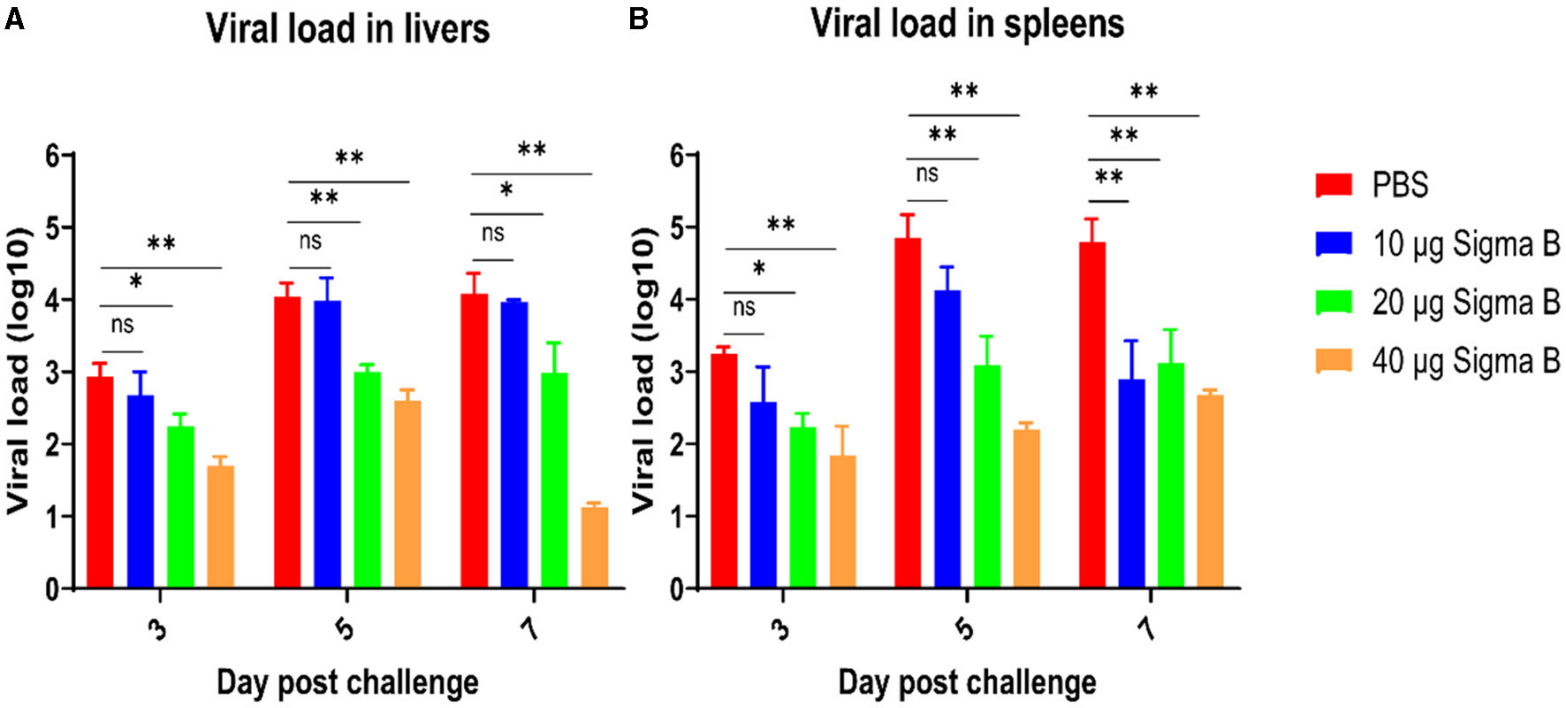
The viral RNA amount in the livers **(A)** and spleens **(B)** after NDRV challenge. Data are represented as means ± SD. ns means no significant, *means *p* ≤ 0.05, **means *p* ≤ 0.01.

The liver and spleen tissue from each group at 3, 5, and 7 DPC were collected for viral load detection. As shown in Figure 8A, the viral load in the livers from the 20-μg MLBE group was markedly lower than that from the PBS group (*p* < 0.05 or *p* < 0.01) at 3, 5, and 7 DPC, and the viral load in the livers from the 40-μg MLBE group was also remarkably lesser than that from the PBS group (*p* < 0.01). The virus load was not significantly different between the PBS and 10-μg MLBE groups. Similarly, the viral load in the spleens from the 20-μg MLBE group was substantially lower than that from the PBS group (*p* < 0.05 or *p* < 0.01) at 3, 5, and 7 DPC, and the viral load in the spleens from the 40-μg MLBE group was also significantly lower than that from the PBS group (*p* < 0.01). The viral load in the spleens from the 10-μg MLBE groups was also considerably lower than that from the PBS group at 7 DPC (*p* < 0.01), and there was no marked variation at 3 and 5 DPC (Figure 8B). These results indicate that MLBE vaccination can reduce the replication of NDRV in the livers and spleens.

## 4 Discussion

NDRV infection in ducklings causes high mortality, resulting in substantial economic losses in the poultry industry in China. Since its first identification, NDRV has widely infected duck flocks in China (25). NDRV is highly different from MDRV, which results in poor cross-protection of the MDRV vaccine against NDRV infection. A commercial live attenuated vaccine of the MDRV CA strain has been used to prevent MDRV. The naturally attenuated NDRV N20 strain also effectively protected against virulent NDRV challenge (19). However, the safety of sN20 requires further evaluation. A subunit vaccine based on the Sigma C protein produced in Sf9 cells was developed, which provided sufficient protection against infection by the Th11 strain (20). However, no commercial vaccine is currently available for preventing NDRV infection. Thus, there is an urgent need to develop a safe and effective vaccine against NDRV for the poultry industry. In the current study, the predicted linear B Cell epitopes of the σB protein of NDRV were expressed in *E. coli* and used as subunit vaccines in ducks. The protective efficacy of the rMLBE was evaluated, with the results suggesting that rMLBE is an attractive candidate for subunit vaccines against virulent NRDV. These results were similar to the research findings on multi-epitope subunit vaccine of influenza viruses (26, 27).

Humoral immunity plays a critical role in disease prevention (28). In this study, the Igγ titers induced by 20 and 40 μg rMLBE were conspicuously higher than those in the PBS group at 7 and 14 days post-inoculation (DPI), while the titers induced by 10 μg rMLBE were significantly higher than those in the PBS group at 14 DPI. These results indicated that the immunization dose was critical for generating Igγ in ducklings. Furthermore, the protective efficacy was evaluated based on the mortality, virus load, and histopathological examinations after challenge with NDRV. Like Igγ titers, immunization with 20 or 40 μg rMLBE provided sufficient protection against NDRV challenge.

In summary, the rMLEB of σB protein prepared using an *E. coli* expression system had good antigenicity and immunogenicity in ducklings, indicating that it can be a vaccine candidate against NDRV infection.

## Data availability statement

The original contributions presented in the study are included in the article/supplementary material, further inquiries can be directed to the corresponding authors.

## Ethics statement

The animal study was approved by the Committee of Animal Experiments of South China Agricultural University, Guangzhou, China (Approval ID: SYXK-2019-0136). The study was conducted in accordance with the local legislation and institutional requirements.

## Author contributions

CL: Conceptualization, Writing – original draft. YC: Methodology, Writing – review & editing. ZY: Writing – review & editing, Supervision. YS: Writing – review & editing, Project administration. QZ: Writing – review & editing, Data curation. HS: Writing – review & editing, Formal analysis. FC: Writing – review & editing, Supervision.
